# Complete Genome Sequence of Multidrug-Resistant Plesiomonas shigelloides Strain MS-17-188

**DOI:** 10.1128/genomeA.00387-18

**Published:** 2018-05-03

**Authors:** Hossam Abdelhamed, Ozan Ozdemir, Hasan C. Tekedar, Mark A. Arick, Chuan-Yu Hsu, Attila Karsi, Mark L. Lawrence

**Affiliations:** a College of Veterinary Medicine, Mississippi State University, Mississippi State, Mississippi, USA; b Institute for Genomics, Biocomputing and Biotechnology, Mississippi State University, Mississippi State, Mississippi, USA

## Abstract

Plesiomonas shigelloides is a Gram-negative bacterium isolated from diverse environments. Here, we describe the complete genome sequence of the multidrug-resistant P. shigelloides strain MS-17-188, isolated from a diseased catfish. Availability of this genome will be beneficial for characterizing the molecular mechanisms of antibiotic resistance in this strain.

## GENOME ANNOUNCEMENT

The genus Plesiomonas is represented by a single homogeneous species, P. shigelloides. It is a Gram-negative, weakly motile, facultatively anaerobic, rod-shaped bacterium that has been classified as a foodborne and waterborne pathogen (1, 2). After many years in the family Vibrionaceae, the genus Plesiomonas is now classified as an unclassified family in the order Enterobacterales (closely related to the family Enterobacteriaceae) (3). P. shigelloides has been isolated from streams, lakes, estuarine waters, crustaceans, reptiles, amphibians, fish, birds, and other mammals (including humans) (4, 5). In humans, it has been implicated in outbreaks of gastroenteritis and diarrhea (6–8). Extraintestinal infections can also occur, causing meningitis, bacteremia, and pseudoappendicitis (9, 10). However, although clinical and epidemiological data imply the role of P. shigelloides in gastrointestinal tract infections, the true pathogenic status of P. shigelloides remains controversial due to a lack of reliable evidence to confirm it as an invasive toxin producer and the lack of a suitable animal model to study its pathogenesis (11).

Plesiomonas shigelloides is the predominant species isolated from intestinal microflora of catfish, catfish pond sediment, and water in the southeastern United States (12, 13). We report here, for the first time, the complete genome sequence of the multidrug-resistant P. shigelloides strain MS-17-188, which was recovered from a diseased catfish in 2017 from the Aquatic Diagnostic Laboratory at the College of Veterinary Medicine, Mississippi State University. P. shigelloides strain MS-17-188 is resistant to gentamicin, tetracycline, oxytetracycline, doxycycline, sulfamethoxazole-trimethoprim, florfenicol, erythromycin, chloramphenicol, streptomycin, azithromycin, penicillin, novobiocin, and spectinomycin.

Illumina (HiSeq X Ten sequencer, Illumina, San Diego, CA, USA) and Nanopore (MinION sequencer, Oxford Nanopore Technologies, Oxford, UK) sequencing methods were used to obtain the complete genome sequence of P. shigelloides strain MS-17-188. The Illumina reads were trimmed and filtered with Trimmomatic (14), and the Nanopore reads were corrected with Canu version 1.6 (15). The chromosome was assembled into a single contig with MaSuRCA version 3.2.4 (16). The average coverages of the P. shigelloides MS-17-188 chromosome and plasmids are summarized in Table 1. Proteins and noncoding RNAs (ncRNAs) were predicted with the NCBI Prokaryotic Genome Annotation Pipeline (PGAP) (17).

**TABLE 1:** Summary of the genome of the P. shigelloides MS-17-188 chromosome and plasmids

Label	GenBank accession no.	Size (bp)	Avg. per-base coverage (×)		GC content (%)
			Illumina	Nanopore	
P. shigelloides MS-17-188 (chromosome)	CP027852	3,505,422	307	91.04	51
pPS-MS-17-188-1 (plasmid)	CP027853	395,858	312.14	78.93	49
pPS-MS-17-188-2 (plasmid)	CP027854	50,109	513.77	122.54	44
pPS-MS-17-188-3 (plasmid)	CP027855	18,970	1,421.62	168.03	62

The complete genome sequence of P. shigelloides MS-17-188 comprised 3,970,359 bp, which included a single contig chromosome (3,505,422 bp) and three circular plasmids (single contig per plasmid), pPS-MS-17-188-1 (395,858 bp), pPS-MS-17-188-2 (50,109 bp), and pPS-MS-17-188-3 (18,970 bp). The three plasmids were not reported in previously published P. shigelloides genomes (strain NCTC10360 [18], strain 302-73 [19], and strains LS1 and LL2 [20]. Among the three plasmids, the pPS-MS-17-188-3 plasmid was found to carry several antibiotic resistance genes. The genome is predicted to encode a total of 3,599 genes, including 3,382 protein-coding genes, 57 pseudogenes, and 160 RNAs (including 40 rRNAs [14, 13, and 13 for 5S, 16S, and 23S, respectively], 116 tRNAs, and 4 ncRNAs). Strain MS-17-188 has ≥97% average nucleotide identity (ANI) with P. shigelloides strains NCTC10360, 302-73, and LS1. This genome may be very useful for determining the molecular mechanisms of antibiotic resistance, including the role of horizontal gene transfer.

### Accession number(s).

The genome sequences of the chromosome and three plasmids reported here have been deposited in GenBank under the accession numbers listed in Table 1.
